# A Study of Professional Quality of Life and Environmental Factors among Pediatric Registered Nurses

**DOI:** 10.1155/2023/5511932

**Published:** 2023-09-04

**Authors:** Kholoud O. Balakhdar, Manal F. Alharbi

**Affiliations:** Maternity and Child Health Department, College of Nursing, King Saud University, Riyadh, Saudi Arabia

## Abstract

**Aim:**

This research study aims to investigate the professional quality of life (ProQOL) among pediatric registered nurses.

**Background:**

Professional quality of life encapsulates two fundamental components, namely, compassion satisfaction and compassion fatigue, which comprises burnout and secondary traumatic stress.

**Methods:**

A descriptive cross-sectional design was used in this study, which included 217 pediatric registered nurses. Self-administered questionnaires compiled sociodemographic and occupational characteristics before measuring Professional quality of life (Version 5) items were used to collect data.

**Results:**

Most respondents were 30 to 35 years of age (65%), female (99%), and hold a diploma certificate (63%). The ProQOL scores for compassion satisfaction was 35.9 (SD = 9.7), compassion fatigue (burnout) was 21.8 (SD = 9.64), and compassion fatigue (secondary traumatic stress) was 23.5 (SD = 9.7). A significant mean difference (*p* < 0.001) were found in the ProQOL level based on environmental factors related to pediatrics and family.

**Conclusion:**

The results of this study show a moderate compassion satisfaction level, a low burnout level, and a moderate secondary traumatic stress level among pediatric registered nurses. Different related environmental factors that can impact the professional quality of life of pediatric registered nurses. *Implications for Nursing Management*. Pediatric nurses will benefit from a supportive work environment, which fosters collaboration and respect among nurses and their pediatric patients' families to improve the health outcomes of pediatric patients. Hospitals should offer specialized training programs that address the unique needs of pediatric nursing, encompassing areas such as child development, communication techniques with children, and age-specific healthcare interventions. This will equip nurses with the necessary skills and knowledge to provide optimal care for pediatric patients. Also, the following support systems, such as regular debriefing sessions and counseling services, are established to help nurses cope with the emotional challenges they may encounter in their work and to foster a collaborative work environment that encourages effective teamwork among nurses and promotes positive relationships between nurses and the families of pediatric patients. This can be achieved by implementing interdisciplinary team approaches, regular team meetings, and open communication channels. In addition, to ensure that pediatric nurses have access to the necessary resources, such as up-to-date medical literature, technological tools, and equipment, to provide the best care possible. Adequate staffing levels should also be maintained to prevent excessive workloads, which can contribute to burnout and decreased job satisfaction.

## 1. Introduction

The healthcare sector in Saudi Arabia plays a significant role in achieving the goals of which focuses on providing high-quality healthcare and creating a vibrant society [1]. One crucial aspect of this sector is pediatric nursing, which is essential for ensuring the well-being of children. However, there is limited awareness in Saudi Arabia regarding the impact of professional quality of life (ProQOL) on pediatric nurses. The quality of professional life is of utmost importance as it directly affects the well-being and job satisfaction of healthcare professionals, including pediatric nurses, high levels of ProQOL can lead to increased compassion satisfaction, positive emotions, and motivation in the work environment, resulting in effective and compassionate care for pediatric patients [2]. On the other hand, a lack of compassion satisfaction among pediatric nurses can lead to negative consequences such as burnout and secondary traumatic stress, compromising the quality of care provided [3].

Hence, there is a need for further research specifically focused on professional quality of life in pediatric nursing, within the context of Saudi Arabia and shedding light on the daily challenges they face and improving their quality of life. The primary aim of this study is to examine the levels of compassion satisfaction and compassion fatigue among pediatric registered nurses. To achieve this, it is crucial to explore the environmental factors that impact ProQOL, including personal-related, work-related, pediatric-related, and family-related factors. Although previous studies have explored the relationship between ProQOL and environmental factors in healthcare providers, there is a gap in knowledge specifically concerning pediatric nursing.

Therefore, the second aim of this study is to investigate the significant mean differences between these factors and professional quality of life in pediatric nursing. By doing so, this study aims to promote the well-being and job satisfaction of pediatric nurses, ultimately leading to improved patient outcomes. The findings of this study can have important implications for enhancing working conditions for pediatric nurses. Regular surveys on ProQOL among pediatric nurses can inform policies and interventions that address their individual needs, contributing to the enhancement of patient outcomes.

## 2. Materials and Methods

### 2.1. Study Design

This quantitative study used cross-sectional to examine the ProQOL levels and various aspects of personal-related environmental factors (PEFs), work-related environmental factors (WEFs), pediatric-related environmental factors (PedEFs), and family-related environmental factors (FEFs) for pediatric RNs [4].

### 2.2. Study Setting and Sample

The study considers pediatric departments via two governmental health institutions included pediatric departments, namely, the Maternity and Children Hospital (MCH) and Heraa General Hospital (HGH) in Makkah, Saudi Arabia. Because there were nurses characterized by different levels of experience and different nationalities who provide basic services to the residents of Makkah and nearby villages, including Hajj-Umrah performers. The target population included the 532 pediatric nurses working in the aforementioned hospitals. It was then necessary to articulate sample criteria, with inclusion criteria enabling the recruitment of registered nurses in different positions working in pediatric departments at the MCH and HGH. Furthermore, nurses were required to speak the English language to communicate efficiently. Meanwhile, healthcare providers were excluded who were working in other departments, pediatric nurses who declined to participate, and internship nurses working in pediatric departments. The sample size was calculated using the SurveyMonkey by Momentive website: https://rb.gy/2si0i. Moreover, assuming values that include a confidence level of 95% to ensure a degree of certainty concerns the selection of populations. A margin of error (e) was allowed (5%). The result was 224, and then 10% was added to the sample size for anticipated missing data and error chance. Consequently, the final sample included 246 pediatric nurses. This study sample allows for valid statistical conclusions and the generalizability of results beyond the sample used. In terms of sampling technique, a controlled quota sampling method has been adopted, with nonprobability sampling used to control data nonrandomly by determining the number of participants in each department. This method would be used to find results quickly and the findings conveniently to administer, minimizing time and cost [4]. First, the participants were divided into different strata based on the hospitals' departments, to identify the number of participants required in each stratum. Then, the proportion (quotas) of participants in each stratum was identified in the target population. After that, these same proportions were applied to the sample size. Consequently, participants were selected from each stratum following the proportions noted in the previous step.

### 2.3. Instrument

The self-reported questionnaire included two parts, one part was about sociodemographic and occupational characteristics and another part corresponded to the ProQOL tool (V5) [2]. In the sociodemographic and occupational characteristics part, the researcher undertook a thorough literature review to identify environmental factors that could operate as independent variables. Personal-related environmental factors (age, gender, sleep medication, and level of education), work-related environmental factors (job position and pediatric department), pediatric-related environmental factors (difficulty communicating with the family of a child and recovery time after the child's death), and family-related environmental factors (number of children living inside the home and marital status) were revealed to influence pediatric RNs' professional quality of life. Questions designed to capture these factors included 10 multiple-choice and dichotomous (closed-ended) questions constructed around the aims of obtaining more factual data, reducing the writing effort demanded of participants, and enabling quick analysis via the tabulation of answers, which increases efficiency. In the ProQOL part, 30 questions were included which were designed to measure the two following introduced domains. To measure CS, items 3, 6, 12, 16, 18, 20, 22, 24, 27, and 30 were included. To measure CF, items concerning burnout, namely, items 1, 4, 8, 10, 15, 17, 19, 21, 26, and 29 of the ProQOL tool and STS, namely, 2, 5, 7, 9, 11, 13, 14, 23, 25, and 28 were included. Responses to these items were given on a 5-point Likert scale, where 1 = never, 2 = rarely, 3 = sometimes, 4 = often, and 5 = very often [2]. In the main research study, the reliability of the ProQOL tool for this study was Cronbach's alpha of 0.89 for CS, 0.83 for BO, and 0.78 for STS. Thus, for all aspects, a Cronbach's alpha of 0.80 or higher is considered especially desirable [4]. Therefore, conceptual equivalence was assessed to check whether the construct has the same meaning across the different groups [5]. Furthermore, there were no notable omissions. In short, the ProQOL tool was appropriate and could be used in this research study. A pilot study has been conducted to assess the research study's feasibility. The participant involved 30 (12%) of the sample size of registered nurses in different hospital pediatric departments. Also, the data would not be used as part of this study. The duration to answer the questionnaires was 10 to 15 min.

### 2.4. Data Collection

Data were collected using a web-based survey as a structured self-report measure. Pediatric nurses were invited to participate in the questionnaire via email sent from the internal communication department of each hospital, with a hypertext link to the Google survey sent to the emails received. Furthermore, a follow-up reminder was sent after five days if a participant partly completed the questionnaire, thanking them for their responses thus far and requesting more cooperation. This method could reduce time and financial costs. This procedure was conducted between January 2023 and March 2023.

### 2.5. Data Analysis

The software Statistical Package for the Social Sciences (SPSS) program (version 29) was used to organize and analyze data. Based on the quantitative analysis, the data analysis combined descriptive and inferential statistics, with descriptive statistics (univariate analysis) via frequency count, percentages, standard deviations (variance), and the central tendency such as mean, for each main variable of the study whether independent or dependent variables. Finally, inferential statistics (bivariate analysis) would be generalized from a sample to a population through parametric testing such as one-way analysis of variance (ANOVA) and independent sample *t*-test. Moreover, results to assess the normal distribution of the data performed the Kolmogorov–Smirnov test and using histogram graphs indicated that the data did conform to a normal distribution. According to the ProQOL manual, reversed answers items 1, 14, 15, and 17, into (1 = 5) (2 = 4) (3 = 3) (4 = 2) (5 = 1). In the end, the sum answers were ≤22 the ProQOL was at a low level, the sum answers were 23–41 the ProQOL was at a moderate level, and the sum answers were ≥42 the ProQOL was at a high level [2]. This research would consider the mean difference significant at the 0.05 level.

### 2.6. Ethical Considerations

Ethical considerations were an essential requirement for this scientific research. First, approval to use the ProQOL tool was obtained from the ProQOL office at the Center for Victims of Torture. Next, the Institutional Review Board of the study setting was obtained (H-02-K076-0123-873 and KSU-HE-22-781). In the end, according to ethical considerations was sent a cover letter to pediatric nurses containing information about the nature, location, and title of the study as well as the ability for them to answer the questionnaire at any time without cost. Furthermore, to address the potential concerns of participants, the letter explained that participation could be voluntary and confidential.

## 3. Results

The questionnaires were e-mailed to 246 potential participants, and 231 participants were agreed to take part in this research study, resulting in a response rate of 93.90% of the sample size. However, during the cleaning of the data, a pattern of missing values was discovered as randomly distributed; it could be due to respondents skipping a question or not being able to provide a response. Moreover, to address this issue and minimize bias in the analysis, it applied a complete-case analysis deletion technique [4]. As a result, the dropout rate was 5.6%, leading to the exclusion of 14 participants. Therefore, the number of questionnaires analyzed was 217, with a response rate of 88.21% out of 246.

In PEFs, characteristics included mostly female nurses (99%, *n* = 214) and the ages were between 30 and 35 years (65%, *n* = 141). Most common was pediatric registered nurses hold a diploma certificate (63%, *n* = 137). Regarding the WEFs 92% (*n* = 199) were nurse specialists or nursing assistants, 63% worked in pediatric intensive care unit (ICU) and pediatric emergency departments (ER) (*n* = 136). For the PedEFs, sixty-one percent (*n* = 132) of participants faced difficulty communicating with the family of a child, while 93% (*n* = 202) did not take recovery time after a child's death and 70% (*n* = 151) married participants with one or more children living at home (64%, *n* = 138) (see Table 1).

To examine the compassion satisfaction and compassion fatigue levels of pediatric registered nurses in Makkah, Saudi Arabia, it was analyzed that descriptive statistics from the data were collected.  Table 2 presents the results of the ProQOL scores for each scale (CS and CF) among the pediatric registered nurses. The mean ± SD for CS level was 35.95 ± 9.77, while for BO and STS levels, and it was 21.88 ± 9.64 and 23.52 ± 9.76, respectively. These results indicate that pediatric registered nurses in Makkah have low levels of BO and moderate levels of CS and STS.

As presented in Table 3, the results showed a significant difference in mean CS levels based on age (*p*=0.04). However, the Tukey HSD post hoc test revealed that there was no significant difference in pairwise comparisons between specific age groups since their corresponding *p* value was above the threshold of 0.05. The results also indicated that gender was significantly related to mean CS and BO levels (*p*=0.002), respectively, but not to mean STS level (*p*=0.40). Also, there were significant mean differences in CS level due to the work in pediatric departments (*p* ≤ 0.001), in BO level (*p* ≤ 0.001), and in STS level (*p* ≤ 0.001); however, the Tukey HSD post hoc test indicates that there was a significant difference between multiple comparisons and department groups since the corresponding *p* value was significant at the 0.05 level. In PedEFs, there were no significant mean differences in CS level due to recovery time after child death (vacations) (*p*=0.36), at BO level (*p*=0.49), and at STS level (*p*=0.17). According to FEFs, there were no significant mean differences in the CS level as a result of the marital status of participants (*p*=0.27), at the BO level (*p*=0.23), and at the STS level (*p*=0.46).

## 4. Discussion

The study focused on examining compassion satisfaction, burnout, and secondary traumatic stress among pediatric registered nurses. Results showed that pediatric RNs had moderate levels of CS, low levels of BO, and moderate levels of STS. These findings align with a previous study conducted in Southern California, which also reported moderate levels of BO, STS, and CS among emergency pediatric nurses [6]. This suggests that the experiences of pediatric RNs in various contexts may lead to similar levels of CS, BO, and STS.

Further analysis of the data highlighted the influence of personal-related environmental factors on ProQOL levels. Specifically, significant gender-related differences were observed in CS and BO levels, with female pediatric RNs more likely to experience BO compared to their male counterparts. This finding contradicts a study conducted in Portugal, which concluded that gender did not affect the quality of work life among pediatric nurses [7]. However, it is important to consider that these gender differences may be influenced by other social and cultural factors, which contribute to varied experiences and well-being among pediatric RNs.

The study also shed light on the relationship between sleep medication use and ProQOL levels among pediatric RNs. It was revealed that the use of sleep medication significantly impacted ProQOL in a negative manner. This finding aligns with other studies that have emphasized the detrimental effects of heavy workloads and insufficient sleep on the well-being and perception of workload among pediatric nurses [6–8]. Considering the consistent findings across these studies, it becomes evident that sleep and medication use play crucial roles in the ProQOL of pediatric RNs. Therefore, healthcare organizations should focus on implementing strategies to promote better sleep hygiene and support the overall mental health and job satisfaction of their nursing staff.

In examining the influence of education levels on ProQOL, it was observed that participants holding bachelor's certificates tended to exhibit higher levels of ProQOL. This finding aligns with a study conducted on pediatric nurses, which revealed that those with master's level education had significantly higher scores of compassion fatigue compared to their counterparts at other education levels in pediatric clinics [9]. However, it is important to consider contrasting findings reported in other studies, such as the study conducted in Portugal that found no significant impact of education level on the quality of work life among pediatric nurses [7]. The variation in results may be attributed to the specific focus of each study, the sample size used, and other contextual factors. Hence, future research endeavors should aim to explore the relationship between education levels and ProQOL in diverse healthcare settings, employing larger sample sizes to enhance the generalizability of the findings.

Regarding age-related differences, the present study did not find significant associations between age and ProQOL levels among pediatric RNs. However, there are discrepancies in the literature regarding the impact of age on ProQOL in this population. For instance, Buckley et al. [3], in a scoping review of existing literature, reported that aged pediatric nurses were affected by burnout levels. The methodological differences between the two studies may explain the contrasting findings, as the present study used quantitative measures, while Buckley et al. [3] conducted a scoping review.

As a result, pediatric RNs' WEFs have a significant impact on their ProQOL levels. The study found that job positions significantly influenced the ProQOL levels of pediatric RNs, with head nurses and charge nurses being affected at the CS level while nurse specialists and nursing assistants were affected by CF levels. Lopez et al. [6] suggested that pediatric emergency nurses need continued support from those in leadership positions to decrease burnout and secondary traumatic stress while continually supporting compassion satisfaction. Therefore, understanding the challenges associated with different pediatric job positions is essential in providing appropriate support and resources to address the challenges associated with different pediatric job positions.

While those in pediatric ICU and ER departments had significant levels of CF, which are components of ProQOL. Previous research conducted in Brazil found that neonatal ICU nurses faced higher levels of burnout compared to other ICU departments, indicating the specific challenges faced by pediatric RNs in this setting [9]. In pediatric ER departments, the fast-paced and unpredictable nature of the work may contribute to constant stress and fatigue, leading to emotional exhaustion and detachment.

Communication with a child's family was identified as a significant factor impacting ProQOL levels among pediatric RNs, whereas no significant mean differences were found regarding recovery time after a child's death. This supports previous research conducted by Koyuncu and Arslan [10]; which indicated that communication problems were reported by 24.2% of pediatric nurses. It is essential to recognize the beneficial implications of interactions with child fatalities on the intervention skills of pediatric nurses but also acknowledge the adverse effects during their working hours. These detrimental effects may extend beyond a single shift and influence subsequent shifts, potentially leading to compassion fatigue. Hence, providing sufficient time and support to address and overcome the negative consequences of child mortality is crucial.

The results of this study indicate that there were significant mean differences between family-related environmental factors and ProQOL levels based on the number of children living in the home. Specifically, pediatric RNs who were living with one or more children had higher levels of compassion satisfaction (CS). This finding is supported by a previous study conducted by Lopez et al. [6]; which found that nurses with one child at home had notably higher CS scores compared to those with two children. On the other hand, no significant mean differences were observed in ProQOL levels based on marital status, consistent with the results of a study conducted by Said et al. [7]; which found no significant difference in the quality of life based on marital status.

In conclusion, understanding the various factors that influence the ProQOL of pediatric RNs is crucial in providing appropriate support and resources. Personal-related environmental, work-related environmental, pediatric-related environmental, and family-related environmental factors all play significant roles in pediatric nurses' ProQOL. By recognizing these factors and addressing their impact, healthcare organizations can better support pediatric nurses in maintaining their professional quality of life.

### 4.1. Limitations of the Work

Web-based questionnaire as a structured self-report, it enabled the examination of a wide range of variables (environmental factors) that may influence these nurses' professional quality of life. In particular, it could make data collection faster and help in reaching a higher response rate. Moreover, the main limitation of using a cross-sectional design in this research study is that it provides only a snapshot of the current situation. Thus, any inferences about relationships based on the data from this study would be limited, and the results may be equivocal. In addition, the primary limitation of using the control quota sampling technique in this research study would be the potential challenges. Because it is a nonprobability sampling technique, the sample set generated through this technique could introduce bias and lack the generalizability often found in probability-based sampling methods and provide limited information that may not reflect the complexity of the pediatric nurses' environment.

## 5. Implications for Nursing Management

QWL is considered an essential component of quality of life, and high QWL is associated with high quality of life. To remain competitive, hospitals must provide nurses with tailored support and resources that address the challenges associated with different pediatric positions as well as by adopting policies that address occupational health and safety, such as ergonomic standards, workload management, and stress reduction programs. Hospital policies should emphasize the development of supportive work environments that foster collaboration and respect among nursing staff, pediatric patients, and patients' families. It would be useful for nurse mangers to provide adequate resources that needed to support nurses in their work and improve the health outcomes of the pediatric patients.

## 6. Conclusion

The pediatric nurses have low levels of burnout and moderate levels of compassion satisfaction and secondary traumatic stress. The gender, sleep medication, level of education, job position, pediatric department, difficulty communication, and number of children were affected on professional quality of life. Otherwise, the age, recovery time, and marital status were not affected by the professional quality of life among pediatric nurses. It recommended further research studies with different research approaches and different low-resource settings such as the remote cities to address the professional quality of life level. The nursing administration in the hospital should provide a healthy environment for pediatric registered nurses, to increase compassion satisfaction levels, keep decreasing burnout level, and manage the secondary traumatic stress level which in turn has a relationship with the professional quality of life level.

## Figures and Tables

**Table 1:** The characteristics of environmental factors of participants in the research study sample (*n* = 217).

Environmental factors		Count	%
*Personal-related environmental factors*			
Age	23–29 years	57	26
	30–35 years	141	65
	Above 35 years	19	9
Gender	Male	3	1
	Female	214	99
Sleep medications	Yes	29	13
	No	188	87
Level of education	Diploma certificate	137	63
	Bachelor's certificate	80	37

*Work-related environmental factors*			
Job position	Head nurse or charge nurse	18	8
	Nurse specialist or nursing assistant	199	92
Work pediatric department	Pediatric ICU or ER departments	136	63
	Pediatric outpatient clinics	18	8
	Other pediatric departments	63	29

*Pediatric-related environmental factors*			
Difficulty communicating with the child family	Yes	132	61
	No	85	39
Recovery time after child death (vacations)	Yes	15	7
	No	202	93

*Family-related environmental factors*			
Number of children living inside the home	Non	79	36
	One and more	138	64
Marital status	Single	54	25
	Married	151	70
	Widowed	2	1
	Divorced	10	5

**Table 2:** Scoring results for professional quality of life levels of participants.

ProQOL	*M*	±SD	Level
CS	35.95	9.77	Moderate^a^
BO	21.88	9.64	Low^b^
STS	23.52	9.76	Moderate^a^

*Note*. ^a^Normal range of moderate level: 23 to 41. ^b^Normal range of low level: ≤22.

**Table 3:** Significant mean difference between difference between environmental factors and professional quality of life (ProQOL) level of pediatric registered nurses.

Environmental factors	ProQOL								
	CS		*P* value	BO		*P* value	STS		*P* value
	*M*	±SD		*M*	±SD		*M*	±SD	
*Personal-related environmental factors (PEFs)*									
Age^a^^*∗∗*^									
23–29 years	47.69	11.59	0.04^*∗*^	51.4	10.1	0.21	50.3	1.33	0.76
30–35 years	50.41	9.23		49.8	9.75		50.0	1.08	
Above 35 years	53.80	9.20		46.8	11.1		48.4	1.12	
*Gender* ^b^									
Male	61.89	3.27	0.01^*∗*^	41.0	1.85	0.002^*∗*^	46.7	5.28	0.40
Female	49.83	9.96		50.1	10.01		50.0	10.04	
Sleep medications^b^									
Yes	58.49	9.41	<0.001^*∗*^	40.8	8.80	<0.001^*∗*^	41.44	8.82	<0.001^*∗*^
No	46.67	8.09		53.5	7.98		53.34	8.31	
Level of education^b^									
Diploma certificate	45.92	7.10	<0.001^*∗*^	53.9	7.22	<0.001^*∗*^	54.6	7.34	<0.001^*∗*^
Bachelor's certificate	56.98	10.40		43.2	10.50		41.9	8.81	

*Work-related environmental factors (WEFs)*									
Job position^b^									
Head nurse or charge nurse	61.0	7.15	<0.001^*∗*^	38.6	7.13	<0.001^*∗*^	41.9	7.29	<0.001^*∗*^
Nurse specialist or nursing assistant	49.0	9.62		51.0	9.59		50.7	9.90	
Work pediatric department^a^									
Pediatric ICU and ER departments	45.1	6.24	<0.001^*∗*^	54.5	6.77	<0.001^*∗*^	55.0	7.20	<0.001^*∗*^
Pediatric outpatient clinic departments	65.2	3.77		34.2	4.09		32.2	2.20	
Other pediatric departments	56.1	10.1		44.5	9.77		44.0	7.09	

*Pediatric-related environmental factors (PedEFs)*									
Difficulty communicating with child family^b^									
Yes	46.5	7.93	<0.001^*∗*^	53.4	8.20	<0.001^*∗*^	53.8	8.39	<0.001^*∗*^
No	55.3	10.5		44.6	10.2		43.9	9.31	
Recovery time after child death (vacations)^b^									
Yes	45.9	14.7	0.36	55.5	14.9	0.49	57.7	13.2	0.17
No	50.2	9.54		49.5	9.45		49.4	9.51	

*Family-related environmental factors (FEFs)*									
Number of children living inside home^b^									
Non	47.6	10.4	0.008^*∗*^	52.0	9.40	0.02^*∗*^	51.8	10.6	0.03^*∗*^
One and more	51.3	9.47		48.84	10.1		48.9	9.47	
Marital status^a^									
Single	47.8	11.7	0.27	52.2	10.4	0.23	51.5	11.5	0.46
Married	50.6	9.14		49.2	9.78		49.6	9.41	
Widowed	54.3	8.03		44.5	10.2		48.2	11.0	
Divorced	51.9	12.1		50.0	9.84		46.8	9.82	

Note. ^a^*F* = one a way ANOVA test, ^b^*t* = test. ^*∗*^Significance two-sided (*p*  < 0.05). ^*∗∗*^No significance in Tukey HSD test (*p*  > 0.05).

## Data Availability

The data that support the findings of this study are available from the corresponding author (KB) upon reasonable request.
